# Supplementary material for: Enhancing Coping and Communication Strategies Following Medical Errors: A Video Case Scenario Workshop for Pediatric Residents
Source: MedEdPORTAL. 2026 Mar 11;22:11581. doi: 10.15766/mep_2374-8265.11581 (PMC12976025; doi:10.15766/mep_2374-8265.11581)
Supplement: Supplementary file 1 — Facilitator Guide.docxCase Scenario and Psychologist Discussion.mp4Psychiatrist Discussion.mp4Preworkshop Questionnaire.docxPostworkshop Questionnaire.docx [file mep_2374-8265.11581-s001.zip › A. Facilitator Guide.docx]

**Appendix A: Facilitator Guide**

Guidelines for the facilitator:

- Inform participants that the workshop is a confidential space for discussion.
- Remind participants of the learning objectives throughout the session.
- Promote active dialogue by asking questions.
- Share relevant personal experiences to foster open conversation.
- Exhibit empathy and honesty in discussions.
- Provide support and offer the opportunity for offline conversations.
- Typically, the workshop lasts about 1 hour with 2 participants, but may extend slightly with 3 participants.

**“Making a medical mistake” facilitator’s debriefing guide (based on the video, Appendix B): First part of the video: patient encounter**

| **Time** | **Introduction, reflection and debriefing by the facilitator** | **Facilitator’s debriefing comments about holding debriefing sessions for a colleague in distress. These comments were made after the comments in the previous column.** |
| --- | --- | --- |
| First 2 minutes  Introduction and objectives for the workshop | Hello, my name is (Name of facilitator), and I am a (facilitator’s title and work description). While being a physician is rewarding, it can sometimes be a challenging profession. The (name of institution) residency program has created a case scenario to provide communication and coping strategies after making a medical mistake. This is a confidential and safe space.  We know each case is unique, and people react differently. As mentioned in my email sent to you before this workshop, when you watch the scenario, think about similar situations you've faced and your feelings, actions, and decisions. We hope these simulations help you reflect on your experiences and support you in future cases.  The objectives of this workshop are as follows:   1. To describe effective communication strategies for disclosing a medical error to patients and their caretakers. 2. To recognize signs and triggers of distress in oneself and colleagues when dealing with adverse patient events. 3. To identify coping strategies after a medical mistake has occurred. 4. Be able to identify when a debriefing session is needed following a medical error or adverse events. 5. Recognize signs that may indicate the need for support and be able to identify the available support systems. |  |
| Next 3-4 minutes | Invite participants to complete the voluntary anonymous pre-workshop survey. Inform them that the responses may be combined and used for research. |  |
| Start the video  Stop the Video at the 1-min, 32 sec- after the intern actor says: “What if he dies” (Discussion for 2-3 minutes) | Please keep in mind that verification of NGT placement varies across institutions, and that not all facilities rely on x-ray confirmation.  In my previous email regarding this workshop, I requested that you consider any medical errors or adverse patient events experienced by you or your colleagues. As this is a safe environment, could each of you briefly share details about those incidents?  The facilitator should be prepared to share their own experiences. By sharing an example of an adverse event from their own training, the facilitator may empower participants to feel safe and share their experiences. | Another objective of our survey is to enable you to conduct a debriefing session for a colleague who has experienced a patient adverse event. If your colleague faces such an event, ensure to find a private area for discussion and inform them that it is a secure environment. |
| Stop the video at the 2 min: 21 secs mark, after the attending says: “I felt bad for a long time”  Discussion: 2-3 minutes | Ask the workshop participants: "What is the attending trying to do when the actor resident is having coping difficulties?" Wait for their responses. Highlight that the attending is validating the resident’s frustration.  Next, ask them: "How would you approach your own cases differently? What emotions did you experience?" Encourage the residents to share their personal experiences.  This method ensures that participants understand the significance of being attuned to their own emotions, recognizing them in others, and subsequently taking appropriate actions. | This is significant when colleagues experience comparable situations. Consider sharing some of your own experiences to acknowledge their emotions.  The facilitator asks the participants: How do you feel after an adverse event? Have you observed signs of distress in colleagues after an adverse event? What are some examples?  Examples given typically include “feel guilty”, “not able to concentrate”, “feeling of not belonging”  The facilitator emphasizes the importance of remembering your own experiences and validating their feelings in a safe space. |
| Stop the video at the 8 min, 56 sec mark when the attending says: “or if you have any questions.”  Discussion: 3 minutes | Ask the workshop participants what they thought about the physician-parent interaction. Ask if they have any positive or negative feedback and what kind of body language or interaction may help during a difficult patient encounter.  Most participants will have comments about body language. The facilitator summarizes the video interaction and participants’ comments and adds her/his own suggestions as needed.   - Being honest and apologizing for the mistake is important. - Communicate exactly what happened and what is known so far, and what happens next. - Be professional and show empathy - Take time and do not rush, so the patient knows you care - Sitting down at the same level as the parent/patient with good eye contact. - Always say what the next step is and that you are going to follow up   The facilitator encourages residents to report adverse events through the incident reporting system and ensures they understand how to report these incidents. | Remember your own experience during a patient encounter and communicate with your colleague about the importance of body language and not rushing when talking to the caretakers.  The facilitator asks participants to review the steps of effective disclosure through an example of a medication error. After the learners’ responses, the facilitator reviews the steps, which include acknowledgment, apologizing, and outlining future steps to rectify the error. |
| Stop the video at the 8-min, 58-second mark, when it says: “A few days later.”  2 minutes | The facilitator expresses that the emotions the resident feels during the patient encounter is normal but if those emotions get prolonged to the point that they interfere with the daily function, the resident should get help.  The facilitator also asks if they are aware of the support systems available at the organization and introduces the participants to some of the available support systems.  In the next part of the video case, the resident seeks help from the psychologist.  If a psychologist is not available, the resident should talk to their attending physician. If that is not possible, they can contact their medical education administrator or human resources for well-being help. Many hospitals and academic centers have well-being resources. | If you recognize a colleague is having a hard time concentrating after a difficult patient encounter, you can support them using the strategies described in this scenario. If the problem persists, you can tell them how they can reach a mental health professional or contact their medical education administration for help. Many schools and hospitals have resources for support. |

**“Making a medical mistake” facilitator’s debriefing guide (based on the video, Appendix B): Second part of the video: psychology visit**

| Start of the video, 9 min mark, when the psychologist's footage begins. | Facilitator: The resident had difficulty concentrating for a few weeks and decided to see a psychologist. This part of the video is about the psychologist and the resident’s encounter. |  |
| --- | --- | --- |
| Stop the video at the 14-min, 30-sec mark, after the intern says: “all those feelings push through again.”  Discussion: 2 minutes | The facilitator asks the participants about how they perceive the conversation so far.  The facilitator summarizes the psychologist’s advice on coping strategies and validating the residents' emotions after adverse events. When the resident in the video shares her feelings of shame, guilt, numbness, and sleep disturbances, the psychologist reassures her that experiencing a dynamic range of emotions is normal.  The facilitator asks the participants if they could remember how they felt after a medical error or an adverse event.  The facilitator acknowledges the feelings and emotions and gives an example of how she/he felt after a patient came back to the emergency department and had been misdiagnosed in a recent hospital admission. | Facilitator: If your colleague experiences coping challenges, you can ask your colleague about their feelings and emotions.  Always acknowledge the emotions and don’t say they should not feel the way they do.  Normalize the emotions: what they are going through is difficult and offer them available support systems. |
| Stop the video at the 17-min, 57 sec mark, after the psychologist says: “changing our perception of what happened.”  Discussion: 2 minutes | The facilitator emphasizes the psychologist’s assessment that, because the resident’s usual coping mechanisms are not working and she is still having difficulty, the response should be regarded as a "traumatic stress response" when usual coping mechanisms are ineffective, rather than simply seeing it as a stressor. If that is the case, seeking support and engaging in verbal processing is important. | The facilitator reminds the participants to be aware of coping difficulties in their colleagues and introduces them to available support systems. |
| End of video | The facilitator reminds the participants when and where to find support systems. | The facilitator reminds participants to inquire about their colleagues' emotions if involved in a patient adverse event. Although we are not psychologists, offering support and validating their feelings is important. Additionally, being aware of institutional support systems is crucial. |

**Making a medical mistake facilitator’s debriefing guide (based on the video, Appendix C): Coping and communication strategies by the psychiatrist**

| **Time** | **The facilitator’s debriefing and reflection comments** | **Facilitator’s debriefing comments about holding debriefing sessions for a colleague in distress** |
| --- | --- | --- |
| Before the video starts | For the next few minutes, one of our psychiatrists will talk about coping strategies and communication skills when dealing with a patient's adverse event | Please remember these strategies to use when holding a debriefing with your colleague. |
| Stop the video at 7-min, 30-sec, when the psychiatrist says: “difficult to do.”  2-3 minutes | The facilitator guide has been revised to include the following important information:  After providing patient care (1st C), it is essential to disclose any mistakes and communicate (2nd C of the 4 C strategy) the details to both the healthcare team and the patient’s family. As residents, you are required to notify your supervisor immediately. When communicating with the family, it is important to acknowledge responsibility, offer an apology, and outline the next steps that will be taken to rectify the adverse event or medical mistake.  Additionally, the facilitator emphasizes that during your orientation, you should have learned about internal processes such as incident reporting. These processes are easily accessible on the hospital computers, and reports can be submitted anonymously. If you have serious concerns, you may contact the Risk Management Office. The facilitator also provides the contact number for Risk Management. | The facilitator asks the participants how they would lead a debriefing session when their colleague is involved in an adverse event. After the participants’ responses, the facilitator reviews the first 2 Cs. The learners were asked to review and practice some language for disclosing a medical error or leading a debrief session after the facilitator coached them during the workshop discussion. |
| Stop the video at its completion  2-minute discussion | The facilitator discusses the importance of the other two Cs: Compassion and Community, which were described as the last two Cs by the psychiatrist as coping strategies that should also be included in debriefing. Compassion: caring for yourself Community: getting support from others.  The facilitator emphasizes the importance of peer support and provides other resources, including the contact information for the psychologist, psychiatrist, as well as well-being resources available at the medical center.  Since psychologists and psychiatrists may not be available at all training sites, the facilitator should be prepared to provide details regarding the setting in which they are leading the session. | After coaching, the facilitator encourages the learners to remember the 4Cs and practice some language for leading a debrief session.  The facilitator encourages participants to be aware of their colleagues' emotions following a medical mistake and to provide support, while also reminding them of the available resources. |
| 1 minute concluding remarks  4 minutes Post-workshop survey | The facilitator eagerly prompts the group for feedback, asking everyone to reflect on their experience of the workshop. The facilitator invites any suggestions on the post-workshop survey on how to enhance future sessions, encouraging open and creative ideas to make the next workshop even more impactful. |  |
