# Supplementary material for: Enhancing Coping and Communication Strategies Following Medical Errors: A Video Case Scenario Workshop for Pediatric Residents
Source: MedEdPORTAL. 2026 Mar 11;22:11581. doi: 10.15766/mep_2374-8265.11581 (PMC12976025; doi:10.15766/mep_2374-8265.11581)
Supplement: Supplementary file 1 — Facilitator Guide.docxCase Scenario and Psychologist Discussion.mp4Psychiatrist Discussion.mp4Preworkshop Questionnaire.docxPostworkshop Questionnaire.docx [file mep_2374-8265.11581-s001.zip › D. Preworkshop Questionnaire.docx]

Pre-Workshop Feedback Survey

This anonymous survey that will take about 5 minutes to complete, will help us understand your challenges in dealing and coping in difficult clinical situations and it will provide us feedback on the effectiveness of our workshop.

**Coping with Adverse Events**

In the past 12 months, have you encountered a Yes

situation where you were directly involved in an No adverse event or making a medical mistake?


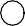

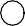


In the past 12 months, have you encountered a Yes

situation where you were indirectly involved in an No adverse event or making a medical mistake?


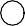

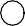


In the past 12 months, have you been involved in a Yes

debriefing session regarding an adverse event or No making a medical mistake?


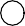

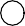


In the past 12 months have you received any training Yes

related to coping after making a medical mistake? No


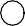

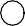


Please explain the training that you received previously:

**Coping with making a medical mistake, please rank the following:**

Strongly Disagree Disagree Agree Strongly Agree

I can cope with the stress
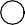

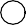

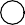

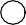
 caused after making a medical

mistake.

I recognize the symptoms of
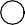

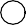

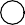

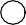
 distress in myself after making a

medical mistake.

I can identify when a debriefing
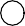

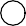

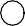

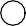
 session for myself is necessary

after making a medical mistake.

I can recognize symptoms of
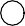

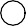

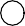

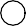
 distress in my colleagues after

making a medical mistake.

I can identify when a debriefing
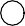

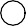

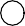

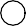
 session is necessary for one of

my colleagues or junior team members after making a medical mistake.

I am comfortable with providing
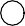

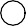

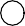

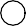
 support for one of my more

junior team members if they are in need after making a medical mistake.

I am aware of the additional
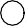

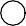

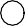

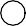
 support systems available to

help me cope after making a medical mistake.

I am familiar with the 4 Cs
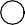

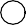

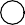

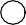
 strategy for coping after making

a medical mistake.

Please describe the barriers in coping with medical mistakes.
