# Supplementary material for: Enhancing Coping and Communication Strategies Following Medical Errors: A Video Case Scenario Workshop for Pediatric Residents
Source: MedEdPORTAL. 2026 Mar 11;22:11581. doi: 10.15766/mep_2374-8265.11581 (PMC12976025; doi:10.15766/mep_2374-8265.11581)
Supplement: Supplementary file 1 — Facilitator Guide.docxCase Scenario and Psychologist Discussion.mp4Psychiatrist Discussion.mp4Preworkshop Questionnaire.docxPostworkshop Questionnaire.docx [file mep_2374-8265.11581-s001.zip › E. Postworkshop Questionnaire.docx]

Post-Workshop Feedback Survey

This anonymous survey that will take about 5 minutes to complete, will help us understand your challenges in dealing and coping in difficult clinical situations, and it will provide us feedback on the effectiveness of our workshop.

**Post-Workshop Feedback Scenario: Coping after making a medical mistake**

Strongly Disagree Disagree Agree Strongly Agree

I can cope with the stress
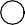

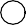

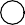

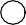
 caused after making a medical

mistake.

I recognize the symptoms of
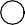

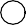

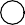

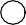
 distress in myself after making a

medical mistake.

I can identify when a debriefing
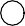

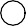

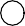

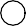
 session for myself is necessary

after making a medical mistake.

I can recognize symptoms of
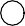

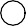

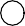

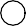
 distress in my colleagues after

making a medical mistake.

I can identify when a debriefing
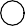

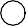

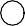

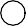
 session is necessary for one of

my colleagues or junior team members after making a medical mistake.

I am comfortable with providing
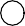

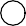

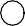

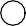
 support for one of my more

junior team members if they are in need after making a medical mistake.

I am aware of the additional
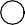

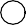

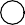

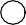
 support systems available to

help me cope after making a medical mistake.

I am familiar with the 4 Cs
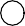

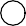

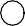

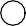
 strategy for coping after making

a medical mistake.

I found this workshop helpful in
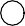

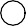

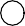

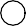
 coping skills after making a

medical mistake.

Please describe the barriers in coping with medical mistakes.

Please tell us if you have any comments about this workshop.

Please tell us if you have any feedback on how to improve this workshop.
