# Supplementary material for: Enhancing Coping and Communication Strategies Following Medical Errors: A Video Case Scenario Workshop for Pediatric Residents
Source: MedEdPORTAL. 2026 Mar 11;22:11581. doi: 10.15766/mep_2374-8265.11581 (PMC12976025; doi:10.15766/mep_2374-8265.11581)
Supplement: Supplementary file 1 — Facilitator Guide.docxCase Scenario and Psychologist Discussion.mp4Psychiatrist Discussion.mp4Preworkshop Questionnaire.docxPostworkshop Questionnaire.docx [file mep_2374-8265.11581-s001.zip › _Educational Summary Report_11581.pdf]

MedEdPORTAL
THE AAMC JOURNAL OF TEACHING AND LEARNING RESOURCES

Original Publication

Open Access

Enhancing Coping and Communication Strategies Following Medical Errors: A Video Case Scenario Workshop for Pediatric Residents

Uma Rao, Celine Payne, Hillary Zieve, MD, Candice Taylor Lucas, MD, MPH, Negar Shekarabi, PsyD, Monisha Vasa, MD, Behnoosh Afghani, MD*

*Corresponding author: bafghani@hs.uci.edu

Abstract

Introduction: Medical errors or adverse events can lead to complex emotions and a decrease in clinical self-confidence in health care workers. Limited training exists to teach residents communication strategies about disclosure, as well as coping and debriefing skills following medical errors. **Methods:** As part of a required rotation for first-year pediatric residents, we developed a 1-hour virtual workshop that included a video case scenario, facilitated discussions, and expert input to teach communication skills and coping strategies following medical errors. The effectiveness of the workshop was evaluated by the first-year pediatric residents through a pre- and postworkshop survey. Participants' self-perceived confidence in their abilities was measured using a 4-point Likert scale before and after the workshop, with pre/post means compared using paired *t* tests. **Results:** All 78 participants completed the pre- and postworkshop surveys. Results of the surveys demonstrated statistically significant increases from pre- to postworkshop in the residents' self-perceived comfort in coping and communication strategies, as well as in identifying the need for a debriefing session. In addition to reporting a wide range of complex emotions, the residents expressed a need for dedicated space, time, and continuous emotional support. **Discussion:** Our results suggest that video case scenarios in combination with facilitated reflection enhanced self-perceived communication and coping strategies following medical errors. Similar workshops can be adapted in other institutions as part of the curricula for first-year pediatric residents as they strengthen their clinical skills and professional identities.

Keywords
Video Case, Coping Strategies, Medical Error, Pediatrics, Adverse Events, Case-Based Learning

Educational Objectives

By the end of this activity, learners will be able to:

1. Describe effective communication strategies for disclosing a medical error or an adverse event to patients and their caretakers.
2. Recognize signs and triggers of distress in oneself and colleagues when dealing with adverse patient events.
3. Identify coping strategies after a medical mistake or adverse event has occurred.
4. Identify when a debriefing session is needed following a medical error or adverse events.
5. Recognize signs that may indicate the need for support and be able to identify the available support systems.

Citation:
Rao U, Payne C, Zieve H, et al. Enhancing coping and communication strategies following medical errors: a video case scenario workshop for pediatric residents. *MedEdPORTAL*. 2026;22:11581.
https://doi.org/10.15766/mep_2374-8265.11581

Introduction

Medical errors or adverse events pose a significant challenge in health care as they can lead to negative consequences in patient care and the emotional well-being of providers.[1] For trainees who are still developing their clinical skills, the impact of such errors can be profound. Medical residents who have experienced a medical error often describe emotional and professional struggles, such as fear of making future mistakes, incompetence, sleep disturbances, and decreased job satisfaction.[2-6] However, most medical education programs lack structured curricula to address the challenges faced by trainees after making a medical mistake, resulting in inadequate preparation to navigate the emotional and professional consequences.[7,8] Inadequate guidance and support in this area can lead to burnout, emotional distress, and career dissatisfaction.[9]

Copyright © 2026 Rao et al. This is an open-access publication distributed under the terms of the Creative Commons Attribution license.

Health care providers who experience emotional distress after an unexpected adverse event or a medical error are known as "second victims," as they have expressed a need for timely and transparent support.[8] Training physicians in coping, debriefing, and communication strategies not only is essential for their well-being, but also promotes trust and safety in the clinical learning environment, and optimizes communication in patient care.[7] Developing healthy coping mechanisms decreases the risk of burnout and enhances their capacity for learning and professional growth.[3] Structured debriefing sessions offer an opportunity to process emotions constructively, reflect on adverse events, identify areas for improvement, and gain valuable long-term insights.[6]

Fostering safe spaces for reflection on medical errors is critical for residents' professional growth. When shown a scenario that included a medical error, most residents identified the error, but fewer than one-half of the residents were able to disclose it effectively.[2] In a recently updated policy statement,[10] the American Academy of Pediatrics emphasizes that pediatricians must disclose medical errors to patients and their families. This policy highlights the need for implementing training programs that equip pediatric physicians with the skills to communicate effectively and compassionately with patients and families following medical errors, and supports institutional priorities that value open communication in error reporting.

Different forms of structured training in medical education, designed to facilitate opportunities for clinicians to observe and debrief with trainees about complex patient interactions, have proven beneficial.[1-3] Resources, including those published in *MedEdPORTAL*, include storytelling and reflection to deal with medical errors or distressing events,[7] or simulation to teach communication skills for disclosing medical errors,[11] and debriefing after distressing events.[12] However, there is a limited literature on programs that integrate workshops into the core curriculum that would help trainees process their emotions after making a medical mistake, help them develop effective coping strategies, and help them communicate appropriately with patients and families in such situations. Recognizing the multifaceted impact of medical errors, our team developed a workshop specifically for pediatric residents, aimed at enhancing their coping abilities and communication skills following such events.

In our report recently published in *MedEdPORTAL*,[13] we demonstrated the effectiveness of video case scenarios in teaching pediatric residents key coping, debriefing, and communication skills following difficult patient encounters. The current report describes the design, implementation, and evaluation of a similar workshop that uses video case scenarios to address the impact of medical errors on participants' well-being. This workshop provides effective communication strategies for disclosing medical errors and coaches residents in coping and debriefing skills after an unexpected adverse event. Our workshop's instructional design is based on several complementary educational theories, particularly Kolb's Experiential Learning Theory,[14] which promotes use of video case scenarios along with facilitated discussion and reflection. We also prioritize psychological safety, enabling residents to discuss errors openly in a supportive learning environment.[15]

By including this workshop as part of the required curricula for first-year pediatric residents, we aimed to ensure that these essential skills are developed early in their training.

Methods

Curriculum Development

As part of the mandatory ACT (Advocacy, Child Abuse, Community Outreach) rotation for first-year pediatric residents, we developed and implemented the Pediatric Educational Discussion Scenarios: Reflect, Improve, Support, and Empower (PEDS-RISE), a 1-hour synchronous virtual workshop to teach trainees communication, debriefing, and coping strategies following medical errors. The workshop, which included a video case scenario, was developed as the result of a collaboration among 2 pediatricians (Hillary Zieve and Candice Taylor Lucas), 1 pediatric hospitalist (Behnoosh Afghani), a psychologist (Negar Shekarabi), and a psychiatrist (Monisha Vasa). During the curriculum development phase, an extensive review of the literature was performed. The scripts of the video scenarios were synthesized based on the information gathered from the literature, as well as the input of our team, who are experts in their respective fields. The video case scenario was role-played by volunteer faculty and medical student actors based on the curated script.

Workshop

The virtual workshop took place during the ACT rotation about 10 times per year. The participants of the workshop included 2–3 first-year residents and a facilitator. A different set of first-year residents participated in the workshop during each rotation. The same facilitator (Behnoosh Afghani) conducted the workshop. Before the workshop, the interns were asked to think about a real case clinical scenario they had encountered previously, and were invited to keep that scenario in mind while watching the video case scenario

Copyright © 2026 Rao et al. This is an open-access publication distributed under the terms of the Creative Commons Attribution license.

during the workshop. At the beginning of the workshop, the facilitator introduced the participants to the goals of the workshop and started the video case scenario. The facilitator stopped the video at certain points to allow time for reflection and discussion among the facilitator and the residents. The reflection and debriefing discussions at different time points of the workshop are detailed in the facilitator's guide (Appendix A).

The workshop on medical mistakes included 2 videos. The first video (Appendix B), along with the reflection discussion, took about 30–35 minutes, while the second video (Appendix C) and the discussion took approximately 20 minutes, summarized as follows:

1. The first video (Appendix B) features an actor portraying a resident grappling with a complex range of emotions after her patient developed aspiration pneumonia following the resident's decision to respect the parent's wishes and cancel a chest radiograph initially ordered to confirm nasogastric tube placement. The second part of this video includes a meeting between a psychologist and the resident, who is having a hard time coping. During this meeting, the psychologist tries to normalize the resident's feelings of guilt and shame. Since the resident's emotions continue to interfere with her daily activities, the psychologist helps the resident to view the event through the lens of trauma, rather than just a stressor, and the resident's lingering emotions as a posttraumatic stress response. The psychologist provides advice on the importance of verbal processing and getting support if the usual coping mechanisms are ineffective.
2. The second video (Appendix C) includes a discussion by a psychiatrist who also validates the emotional response of the resident and emphasizes the fact that making mistakes is inevitable at some point in our careers, as we are all human beings. She provides a framework for communication and coping strategies. These strategies are summarized as the 4Cs strategies for coping after making a medical mistake:
   - Care: Make sure the patient gets appropriate care after the adverse event promptly.
   - Communicate: Disclose the medical error to the team and family in an honest way.
   - Compassion: Compassionately communicate with the patient and their family, and pay attention to your own self-care.
   - Community: Lean on the community around you, and get support from people at work or people close to you.

Throughout the video case scenario, the facilitator stopped the video at different points (a detailed timeline of the designated pause points is shown in Appendix A) and promoted active dialogue by sharing relevant personal experiences to foster open conversation and reflection. In addition, during videos by the psychologist and psychiatrist, the facilitator emphasized the takeaway points raised by them through personal examples, reflection, and discussion with the resident participants.

Evaluation Methods

To evaluate the workshop outcomes, we distributed a pre- and postworkshop anonymous survey (Appendices D and E). The survey asked about some baseline characteristics and measured the residents' self-perceived communication and coping skills before and after the workshop. We employed paired *t* tests to compare the pre- and postworkshop responses, with ratings based on 4-point Likert scales (1 = *Strongly disagree*; 4 = *Strongly agree*). A *P* value of less than or equal to .05 was considered statistically significant. We created the survey based on similar survey instruments that have been published.[16] The survey underwent multiple rounds of iterative review process with multiple rounds of feedback and revision by the pediatricians (Behnoosh Afghani, Hillary Zieve, and Candice Taylor Lucas) and a psychologist (Negar Shekarabi). In addition, the survey asked an open-ended question: "Please describe the barriers in coping with medical mistakes." Two authors (Behnoosh Afghani and Uma Rao) independently reviewed the responses and organized them into categories to facilitate descriptive analysis of the data.

Further assessment was conducted during the reflections, as several strategies, including communication, debriefing, and coping techniques, were explored through the facilitator's guidance. The learners were encouraged to review and practice specific language for disclosing a medical error or leading a debrief session, following coaching from the facilitator during the workshop discussion.

Results

A cohort of 78 first-year pediatric residents participated in our workshop starting in August 2022 through August 2025. A comparison of pre- and postworkshop quantitative results revealed significant improvements in residents' self-reported outcomes. Postworkshop survey scores indicated that participants had enhanced coping skills, increased self-awareness, and a greater recognition of the need for debriefing. Additionally, residents reported feeling more capable of supporting their peers and demonstrated increased awareness of available support systems and communication strategies following medical errors (Table 1).

Copyright © 2026 Rao et al. This is an open-access publication distributed under the terms of the Creative Commons Attribution license.

Table 1. Survey Responses Before and After Participation in the Workshop on Coping and Communication Strategies Following Medical Errors ($N = 78$)

Survey Statement[a]	Pre *M* (*SD*)	Post *M* (*SD*)	*P*
I can cope with the stress caused after making a medical mistake.	2.6 (0.5)	3.1 (0.4)	<.0001
I recognize the symptoms of distress in myself after making a medical mistake.	3 (0.5)	3.3 (0.5)	.0001
I can identify when a debriefing session for myself is necessary after making a medical mistake.	2.7 (0.7)	3.3 (0.5)	<.0001
I can recognize symptoms of distress in my colleagues after making a medical mistake.	2.8 (0.6)	3.2 (0.5)	<.0001
I can identify when a debriefing session is necessary for one of my colleagues or junior team members after making a medical mistake.	2.5 (0.6)	3.2 (0.5)	<.0001
I am comfortable with providing support for one of my more junior team members if they are in need after making a medical mistake.	2.6 (0.7)	3.2 (0.6)	<.0001
I am aware of the additional support systems available to help me cope after making a medical mistake.	2.2 (0.6)	3.3 (0.5)	<.0001
I am familiar with the 4 Cs strategy for coping after making a medical mistake.	1.8 (0.5)	3.5 (0.5)	<.0001

[a] First-year pediatric residents rated responses on a 4-point scale (1 = *Strongly disagree*; 4 = *Strongly agree*).

Table 2 presents example excerpts from the residents' responses to the free-text question: "Please describe the barriers in coping with medical mistakes." Each excerpted comment is accompanied by its corresponding descriptive category. It is important to note that some statements may fall into multiple categories, while a few may not clearly align with any of the 6 identified categories. Residents reported facing significant emotional challenges, such as guilt as well as limited support, time constraints, and difficulties in communicating with the families, as the main barriers.

Discussion

Our study demonstrates that a targeted workshop can significantly improve residents' self-perceived familiarity with communication, coping, and the need for debriefing following medical mistakes. These findings align with previous research highlighting the importance of structured interventions in enhancing health care professionals' ability to manage the aftermath of adverse events.[4,10,11,17] Our video case scenario and the subsequent reflections, developed through close collaboration among pediatricians, a psychologist, and a psychiatrist, provided a powerful tool to address the emotional and professional challenges that arise after a medical mistake. This approach fills a significant gap that has been identified in current *MedEdPORTAL* publications.

Previous studies have used role-play and self-reflection,[7] small-group trainings through simulation,[11] or storytelling.[7] Our approach builds upon existing methods by emphasizing individual resident emotional processing through the analysis of a video-based scenario. In small-group discussions with 2–3 residents, the facilitator uses a video case study as a catalyst to explore the participants' personal experiences with medical errors. This format aligns with the psychological safety theory[15] and Kolb's Experiential Learning Theory,[14] intentionally creating a space for reflection and shared learning, which encourages residents to feel safe and explore their emotions and coping mechanisms.[18,19] Our workshop is unique as it provides an opportunity for the learners to witness a therapy session with a psychologist. The psychologist and the facilitator support resident engagement by validating the trainees' emotional responses to medical mistakes, normalizing their experiences, and enabling them to understand and manage their emotions effectively. The psychiatrist complements this by offering evidence-based coping and communication strategies to equip the residents with practical tools for navigating the consequences of medical errors. This multifaceted approach, which incorporates both theoretical scenarios and real-world experiences, coupled with expert guidance, not only enhances individual growth and emotional intelligence but also contributes to a culture of transparency, continuous improvement, and patient safety in clinical practice.

Our descriptive findings revealed that resident physicians experience a spectrum of complex emotions when confronting medical mistakes. As expressed by our residents, emotions such as "concern for how others will perceive you, isolation, shame," "processing the guilt," as well as "shame around making a mistake," can trigger rumination cycles leading to isolation and disconnection.[3,6] These emotions reflect the well-documented "second victim" phenomenon, where health care workers, especially trainees, experience moral distress, diminished clinical confidence, and reduced self-esteem after adverse events.[6]

Feelings of guilt remained a prominent theme after the workshop, demonstrating that a brief workshop alone may be insufficient to resolve these deeper and more complex emotions. Ongoing support and more comprehensive interventions to address the emotional challenges associated with medical errors are needed. Additionally, residents frequently cited challenges in obtaining support and challenges in communicating with families as major barriers. The ongoing presence of emotional challenges before and after the workshop can be attributed to the workshop's focus on the intricacies of honest and open communication, along with

Copyright © 2026 Rao et al. This is an open-access publication distributed under the terms of the Creative Commons Attribution license.

Table 2. Sample Resident Responses by Category to a Free-Text Question Before and After Participation in the Workshop on Coping and Communication Strategies Following Medical Errors[a]

Category	Preworkshop Comments	Postworkshop Comments
Guilt	"I hate feeling like I hurt my patients and it affects my whole mood."<br>"Understanding that you may have seriously hurt a patient."<br>"Guilt of what I could have done, an unfortunate moment that is hard to forget."	"Self-blame, feelings of incompetence."<br>"Processing the guilt afterwards."<br>"Feeling guilty about making a medical mistake."
Imposter syndrome	"Feeling like a failure, hard to admit mistake."<br>"Feeling like I am not good enough."<br>"Personal barriers include imposter syndrome, low confidence, guilt."	"Embarrassment, feeling like a fraud."<br>"Feelings of inadequacy. Guilt of potential harm."<br>"Personal stressors such as guilt, personal trauma, imposter syndrome, not having a good support system."
Fear of repercussion	"Fear of litigation or punishment."<br>"It feels like we will 'get in trouble' if admitting to a mistake."<br>"Not wanting to admit fault, medico-legal repercussions."<br>"When there is non-compassionate discipline from admin or superiors exacerbating the issue."<br>"Not wanting to admit the mistake to your team members, feeling judged for making a mistake, backlash/retaliation from either the parents/patient or the attendings."	"Not wanting to tell the parents the truth for fear of retaliation/being sued."<br>"Being afraid of the consequences associated with the mistakes."
Time constraint	"We often don't have time to discuss mistakes."<br>"Time while doing work. We need more time off."<br>"Not having enough time to properly cope with the mistake or take the steps to ensure it doesn't happen again through new policies or developments."	"Time while doing work."<br>"Time-constraints, overall workload."<br>"Busy Schedules, hard to make appointments to debrief with healthcare professionals."
Communication/support	"Feelings of frustration and anger at self, feelings of inability to act, feeling inadequate, figuring out how to talk with the parents and family after medical mistake."<br>"Difficult to disclose to families."<br>"Not wanting to admit the mistake to your team members, feeling judged for making a mistake, backlash/retaliation from either the parents/patient or the attendings."<br>"Communication, support, resolution."	"Getting the courage to face the family after making the mistake."<br>"Communication, team dynamic, relationship with patient/parents."<br>"Feeling comfortable asking others to discuss the mistake that was made."<br>"…another barrier is recognizing when I need additional support and seeking help from a therapist."<br>"Access to consistent mental health resources, systems that scapegoat individuals."
Coping	"Support & coping strategies afterwards."<br>"Not knowing the resources and support available. Feeling ashamed to talk about it."<br>"Difficulty identifying and addressing those feelings."<br>"Having the time to process and cope. Finding people to debrief with."<br>"I think I usually just try to learn from the mistake and start blaming myself."<br>"How to rectify the situation and deal with feelings of guilt."	"Having to continue to work through stress and not wanting to burden the back-up system to take the time off needed for self-care."<br>"Emotional stressors, everyone's response to coping with such mistakes is likely different, lack of experiencing medical mistakes and what to do when they are experienced. This lecture laid a nice framework!"

[a]First-year pediatric residents ($N = 78$) responded to the following question: "Please describe the barriers in coping with medical mistakes."

the significance of seeking professional mental health support when emotional distress impacts daily life. It is possible that the increased awareness of these issues during the workshop likely prompted residents to recognize their significance more acutely. Residents' postworkshop comments support this hypothesis. For example, 1 participant noted, "Another barrier is recognizing when I need additional support and seeking help from a therapist." Another resident highlighted systemic issues, citing "access to consistent mental health resources" and "systems that scapegoat individuals" as additional barriers. Based on these responses, it is crucial to allocate dedicated time, space, and resources for health care professionals who require mental health support services following medical errors.

Time constraints also emerged as a significant barrier in our study, with residents citing "having the time to process and cope" as a major challenge in dealing with medical mistakes. Notably, even after our workshop, residents expressed that the time needed to address the consequences of medical errors remained a persistent issue, indicating that our intervention alone is insufficient to overcome time-related constraints. Consistent with previous studies, our findings underscore the need for additional systemic interventions to mitigate the pressures of a demanding clinical environment, creating dedicated space and time for error disclosure, reflection, and debriefing.[8,10,11]

Our workshop addresses a critical need identified both in our findings and in existing literature[2]: educating trainees on effective communication strategies for disclosing medical errors. A central theme that emerged from our study was the challenge of communication and disclosure. As expressed by our residents, their concerns of "getting the courage to face the family after

Copyright © 2026 Rao et al. This is an open-access publication distributed under the terms of the Creative Commons Attribution license.

making a mistake" and "figuring out how to talk with the parents and family after a medical mistake" highlight the importance of developing robust communication skills in these sensitive situations. Our findings align with those of Coffey et al, who reported that, although more than 90% of pediatric residents could identify medical errors, only 40% would disclose them.[2] Their research revealed that disclosure decisions are heavily influenced by contextual factors, including hierarchical position and team dynamics, which may explain the communication anxiety expressed by our residents.[2] The American Academy of Pediatrics emphasizes the need for structured error disclosure education,[10] a stance supported by findings that fewer than one-half of residents have received formal teaching on disclosure. This significant gap in medical education is precisely what our workshop aims to address. In the postworkshop survey, fewer residents reported fear of repercussion or retaliation as a barrier to error disclosure. This reduction in fear may be attributed to our workshop's emphasis on effective communication strategies and the importance of honest disclosure of medical errors. Our focus on these areas appears to have helped residents better navigate these concerns.

While our workshop demonstrates a promising approach to addressing the challenges residents experience following medical errors, we acknowledge several limitations in our current study. Our findings are based on data from a single institution, potentially limiting external generalizability. Moreover, at present, we conduct only immediate postworkshop evaluations, which may not capture long-term effects. In addition, residents' survey responses may be subject to social desirability bias, and may not accurately reflect behaviors in real-world situations.

To strengthen future iterations of this research, we plan to implement longitudinal assessments to evaluate the longevity of attitudinal and behavioral changes regarding medical errors. Expanding to multicenter studies would enhance generalizability and account for institutional variations. Incorporating objective measures of performance in addition to self-reported data would also provide a more comprehensive evaluation. To further enhance the effectiveness of our approach, we propose incorporating a hands-on workshop where residents can practice disclosure and debriefing skills and receive real-time feedback. This addition would reinforce the application of learned skills in a supportive environment.

While immediate access to mental health professionals following medical incidents may not always be feasible in clinical settings, our comprehensive workshop model intentionally equips residents with essential coping strategies for processing experiences with medical errors. This proactive approach bridges the gap between ideal support and practical constraints, and fosters resilience and self-efficacy. Our workshop aims to instill effective communication, coping, and debriefing strategies that residents can employ when they or their colleagues face guilt, incompetence, and lack of belonging following a medical mistake. By providing these tools, we seek to normalize a culture that encourages open communication and mutual support within the medical community. We believe that our innovative workshop can be readily integrated into residency curricula to support professional development and address a critical gap in medical education by providing residents with essential tools to navigate the complex emotional and communication challenges associated with medical errors.

Appendices

A. Facilitator Guide.docx
B. Case Scenario and Psychologist Discussion.mp4
C. Psychiatrist Discussion.mp4
D. Preworkshop Questionnaire.docx
E. Postworkshop Questionnaire.docx

All appendices are peer reviewed as integral parts of the Original Publication.

Uma Rao: Medical Student, University of California, Irvine, School of Medicine

Celine Payne: Medical Student, University of California, Irvine, School of Medicine

Hillary Zieve, MD: Health Sciences Assistant Clinical Professor, Department of Pediatrics, University of California, Irvine, School of Medicine and Rady Children's Health, Orange County

Candice Taylor Lucas, MD, MPH: Health Sciences Clinical Professor, Department of Pediatrics, University of California, Irvine, School of Medicine

Negar Shekarabi, PsyD: Clinical Psychologist, Faculty/Staff Support Services, Benefits & Total Rewards, UC Irvine Enterprise

Monisha Vasa, MD: Clinical Psychiatrist, Volunteer Assistant Clinical Professor, Department of Family Medicine, University of California, Irvine, School of Medicine

Behnoosh Afghani, MD: Health Sciences Clinical Professor, Department of Pediatrics, University of California, Irvine, School of Medicine and Rady Children's Health, Orange County; ORCID: https://orcid.org/0000-0002-1556-500X

Copyright © 2026 Rao et al. This is an open-access publication distributed under the terms of the Creative Commons Attribution license.

Acknowledgments
We thank the University of California, Irvine (UCI)/Rady Children's Health, Orange, CA, Pediatric Residency Program, for supporting this project, the UCI Health Family Health Center, in the city of Santa Ana, for providing space for videotaping case scenarios, as well as Trinidad Alcala-Arcos, a UCI School of Medicine medical student. We are also grateful to Josh Zieve for his videography and editing.

Disclosures
None to report.

Funding/Support
Dr. Behnoosh Afghani reports this work was funded in part by an M-Power mini-grant from the University of California, Irvine, School of Medicine and the Song-Brown grant from the California Department of Health Care Services.

Ethical Approval
The University of California, Irvine, School of Medicine Institutional Review Board reviewed this project.

References

1. Liukka M, Steven A, Vizcaya Moreno MF, et al. Action after adverse events in healthcare: an integrative literature review. *Int J Environ Res Public Health*. 2020;17(13):4717. https://doi.org/10.3390/ijerph17134717
2. Coffey M, Thomson K, Tallett S, Matlow A. Pediatric residents' decision-making around disclosing and reporting adverse events: the importance of social context. *Acad Med*. 2010;85(10): 1619-1625. https://doi.org/10.1097/ACM.0b013e3181f046a6
3. Fatima S, Soria S, Esteban-Cruciani N. Medical errors during training: how do residents cope?: A descriptive study. *BMC Med Educ*. 2021;21(1):408. https://doi.org/10.1186/s12909-021-02850-1
4. Kronman AC, Paasche-Orlow M, Orlander JD. Factors associated with disclosure of medical errors by housestaff. *BMJ Qual Saf*. 2012;21(4):271-278. https://doi.org/10.1136/bmjqs-2011-000084
5. Waterman AD, Garbutt J, Hazel E, et al. The emotional impact of medical errors on practicing physicians in the United States and Canada. *Jt Comm J Qual Patient Saf*. 2007;33(8):467-476. https://doi.org/10.1016/S1553-7250(07)33050-X
6. Wu AW, Steckelberg RC. Medical error, incident investigation and the second victim: doing better but feeling worse? *BMJ Qual Saf*. 2012;21(4):267-270. https://doi.org/10.1136/bmjqs-2011-000605
7. Adkins S, Reynolds P, Rabah K, Flowers S. Medical error: Using storytelling and reflection to impact resident error response factors. *MedEdPORTAL*. 2024;20:11451. https://doi.org/10.15766/mep_2374-8265.11451
8. Edrees HH, Paine LA, Feroli ER, Wu AW. Health care workers as second victims of medical errors. *Pol Arch Med Wewn*. 2011;121(4):101-108. https://doi.org/10.20452/pamw.1033
9. West CP, Huschka MM, Novotny PJ, et al. Association of perceived medical errors with resident distress and empathy: a prospective longitudinal study. *JAMA*. 2006;296(9):1071-1078. https://doi.org/10.1001/jama.296.9.1071
10. Sigman L, Turbow R, Neuspiel D, Kim JM; Committee on Medical Liability and Risk Management; Counsil on Quality Improvement and Patient Safety. Disclosure of adverse events in pediatrics: policy statement. *Pediatrics*. 2025;155(4):e2025070880. https://doi.org/10.1542/peds.2025-070880
11. McDonough KA, White AA, Odegard PS, Shannon SE. Interprofessional error disclosure training for medical, nursing, pharmacy, dental, and physician assistant students. *MedEdPORTAL*. 2017;13:10606. https://doi.org/10.15766/mep_2374-8265.10606
12. McDermott A, Brook I, Ben-Isaac E. Peer-debriefing after distressing patient care events: a workshop for pediatric residents. *MedEdPORTAL*. 2017;13:10624. https://doi.org/10.15766/mep_2374-8265.10624
13. Payne C, Zieve H, Lucas CT, et al. Pediatric Educational Discussion Scenarios: Reflect, Inspire, Support, and Empower (PEDS-RISE)—A difficult patient encounter video scenario. *MedEdPORTAL*. 2025:21:11522. https://doi.org/10.15766/mep_2374-8265.11522
14. Kolb DA. *Experiential Learning: Experience as the Source of Learning and Development*. Second edition. Pearson Education; 2015.
15. Edmondson AC. *The Fearless Organization: Creating Psychological Safety in the Workplace for Learning, Innovation, and Growth*. John Wiley & Sons, Inc.; 2019.
16. Govindan M, Keefer P, Sturza J, Stephens MR, Malas N. Empowering residents to process distressing events: a debriefing workshop. *MedEdPORTAL*. 2019:15;10809. https://doi.org/10.15766/mep_2374-8265.10809
17. Wykowski JH, Merel S, Starks H, et al. An embedded curriculum to teach critical incident debriefing to internal medicine residents. *J Grad Med Educ*. 2024;16(1):59-63. https://doi.org/10.4300/JGME-D-23-00273.1
18. Hardie P, O'Donovan R, Jarvis S, Redmond C. Key tips to providing a psychologically safe learning environment in the clinical setting. *BMC Med Educ*. 2022;22(1):816. https://doi.org/10.1186/s12909-022-03892-9
19. Ali R, Afzal A, Shakil S, et al. Transforming education: case-based integrated learning development and implementation—a mixed methods study at a private medical college. *J Adv Med Educ Prof*. 2025;13(1):49-58. https://doi.org/10.30476/jamp.2024.104285.2048

Received: September 3, 2025
Accepted: January 9, 2026
Published: March 11, 2026

Copyright © 2026 Rao et al. This is an open-access publication distributed under the terms of the Creative Commons Attribution license.
